# Low-cost plasmonic solar cells prepared by chemical spray pyrolysis

**DOI:** 10.3762/bjnano.5.249

**Published:** 2014-12-12

**Authors:** Erki Kärber, Atanas Katerski, Ilona Oja Acik, Valdek Mikli, Arvo Mere, Ilmo Sildos, Malle Krunks

**Affiliations:** 1 Laboratory of Thin Film Chemical Technologies, Department of Materials Science, Tallinn University of Technology, Ehitajate tee 5, 19086 Tallinn, Estonia; 2 Chair of Semiconductor Materials Technology, Department of Materials Science Tallinn University of Technology, Ehitajate tee 5, 19086 Tallinn, Estonia; 3 Institute of Physics, University of Tartu, Riia 142, 51014, Tartu, Estonia

**Keywords:** Au nanoparticles, chemical spray pyrolysis, extremely thin absorber, plasmon resonance, solar cell

## Abstract

Solar cells consisting of an extremely thin In_2_S_3_/CuInS_2_ buffer/absorber layer uniformly covering planar ZnO were prepared entirely by chemical spray pyrolysis. Au nanoparticles (Au-NPs) were formed via thermal decomposition of a gold(III) chloride trihydrate (HAuCl_4_·3H_2_O) precursor by spraying 2 mmol/L of the aqueous precursor solution onto a substrate held at 260 °C. Current–voltage scans and external quantum efficiency spectra were used to evaluate the solar cell performance. This work investigates the effect of the location of the Au-NP layer deposition (front side vs rear side) in the solar cell and the effect of varying the volume (2.5–10 mL) of the sprayed Au precursor solution. A 63% increase (from 4.6 to 7.5 mA/cm^2^) of the short-circuit current density was observed when 2.5 mL of the precursor solution was deposited onto the rear side of the solar cell.

## Introduction

The cost of solar cells scales with the complexity of the technology involved as well as the price and volume of the semiconductors used (but in particular, the absorber material). The use of very thin absorber layers in solar cells requires adoption of various light trapping techniques to take advantage of the smaller absorbing volume. The use of mesoporous TiO_2_ or ZnO nanorods provides increased surface area of the absorber, while the introduction of metal nanoparticles allows photons to be captured via plasmonic effects [1–4]. This work attempts to utilize the advantages of the plasmon effect, while providing a technologically simple method for solar cell production.

Chemical spray pyrolysis (CSP) is a simple method to produce thin semiconductor oxide- and sulphide layers and metal nanoparticles (NPs) via thermal decomposition of metal precursor salts. CuInS_2_ (CIS) is a semiconductor material with a band gap of 1.5 eV that is often used as a photovoltaic absorber. Previously published, related work by our research group regarding CIS-based solar cells includes: the synthesis and properties of CIS [5–6], application of CIS in extremely thin absorber solar cells based on ZnO nanorods [7], the thermal decomposition of a HAuCl_4_·3H_2_O precursor solution for Au-NP formation [8], and CIS/Au-NP and Au-NP/CIS composite layers prepared by spraying on glass [9]. In the composite layers, the Au-NPs assist photon absorption in the CIS absorber in the wavelength region of 500–800 nm [9].

Increased photocurrent due to the plasmonic effects of NPs have been demonstrated, for example, for thin film Si solar cells [3], polymer cells [10–11], dye-sensitized cells [12–13] and for solar cells that use ultrathin inorganic absorber layers [14]. However, the use of an in-line spray method for the deposition of the solar cell, including the plasmonic NPs within the cell, has not yet been published.

In the present study, Au-NP layers are deposited by CSP at various stages of the solar cell preparation. This work investigates which locations within the solar cell are optimal for the deposition of Au-NPs in order to increase the photocurrent in the sprayed solar cell.

## Experimental

Using commercially available, ITO-covered glass as a substrate, Au-NPs were deposited onto the ITO layer (ITO/Au-NP/ZnO/In_2_S_3_/CuInS_2_) or on top of the absorber layer (ITO/ZnO/In_2_S_3_/CuInS_2_/Au-NP). Details regarding the ITO/ZnO/In_2_S_3_/CuInS_2_ solar cell preparation by spray pyrolysis can be found elsewhere [15].

For the deposition of the Au-NP layer, gold(III) tetrachloride trihydrate (HAuCl_4_∙3H_2_O, 99.9%, Aldrich) was dissolved in deionized water at a concentration of 2 mmol/L and used as a precursor. The solution was pneumatically sprayed through air onto a substrate with a surface temperature of 260 °C. The solution volume was varied from 2.5 to 10 mL and the solution feeding rate was 1 mL/min.

Current–voltage scans of the solar cells were used to obtain the principal characteristics of the solar cells: voltage at open-circuit condition (*V*_OC_), current density at short-circuit condition (*J*_SC_), the fill factor (FF) and the conversion efficiency (η).

The total reflectance spectra of the solar cells were measured in the wavelength range of 300–1500 nm on a Jasco V-670 spectrophotometer equipped with an integrating sphere.

The external quantum efficiency (EQE) of the solar cells was measured in the range of 300–1000 nm on a Newport Oriel kit that contains a 300 W Xe lamp, high-resolution monochromator (Cornerstone 260), digital dual-channel lock-in detector (Merlin), and a calibrated silicon reference detector. The Xe lamp is a light source which simulates the conventional AM1.5 spectrum for testing solar cells. The dispersed light from the Xe lamp (incident on the solar cell as monochromatic light) was optically chopped at 30 Hz. EQE is defined as the number of collected charge carriers per incident photon. The EQE is a unitless characteristic (EQE < 1) given by:

[1]
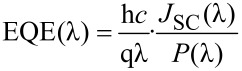


where *J*_SC_(λ) (A·m^−2^) is the spectrally resolved short-circuit current of the solar cell, *P*(λ) (W·m^−2^) is the calibrated light intensity incident on the solar cell, and h*c*/qλ is the energy (eV) of a photon as function of wavelength λ.

For visualization of the morphology of the cross-section of the solar cells, a Zeiss HR FESEM Ultra 55 scanning electron microscope (SEM) at operating voltage of 4 kV was used. The electron beam induced current (EBIC) mode of the SEM was used to map the local electronic activity of the solar cells.

## Results and Discussion

**Figure 1:**
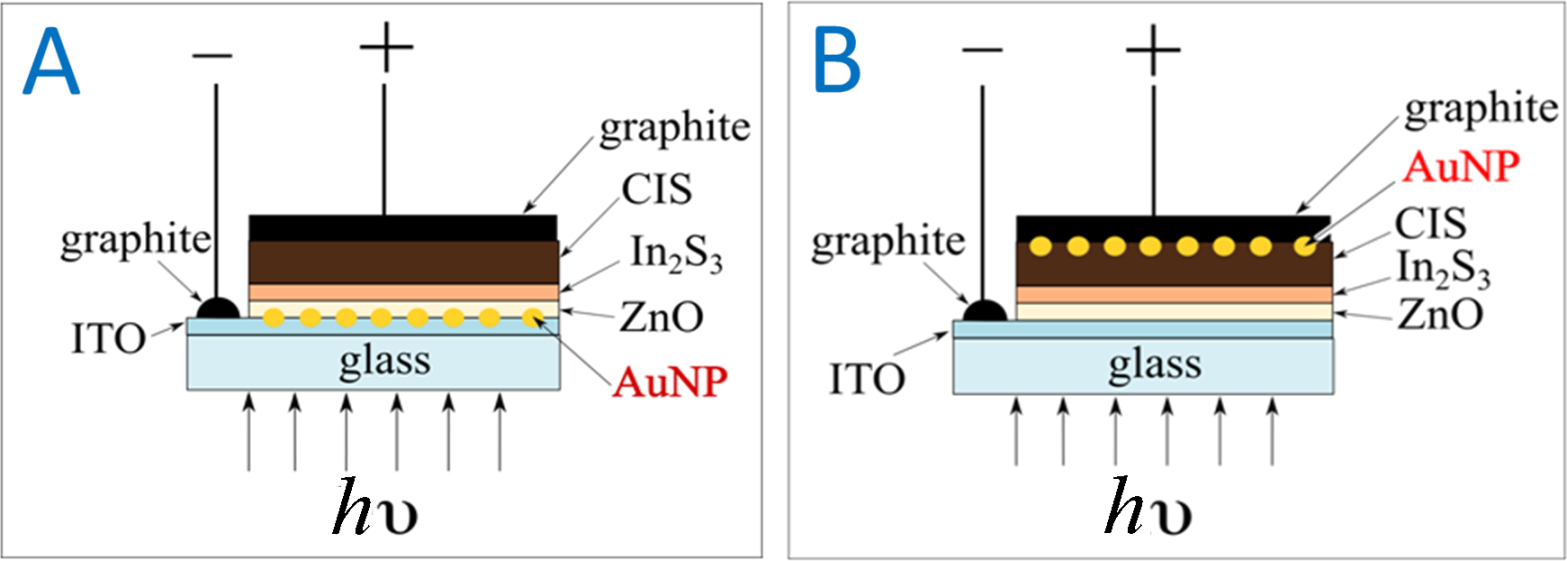
Sketch of the cross-section of the Au-NP/ZnO/In_2_S_3_/CuInS_2_ solar cell (A), and of the ZnO/In_2_S_3_/CuInS_2_/Au-NP solar cell (B), all layers prepared by chemical spray using ITO/glass substrates.

**Figure 2:**
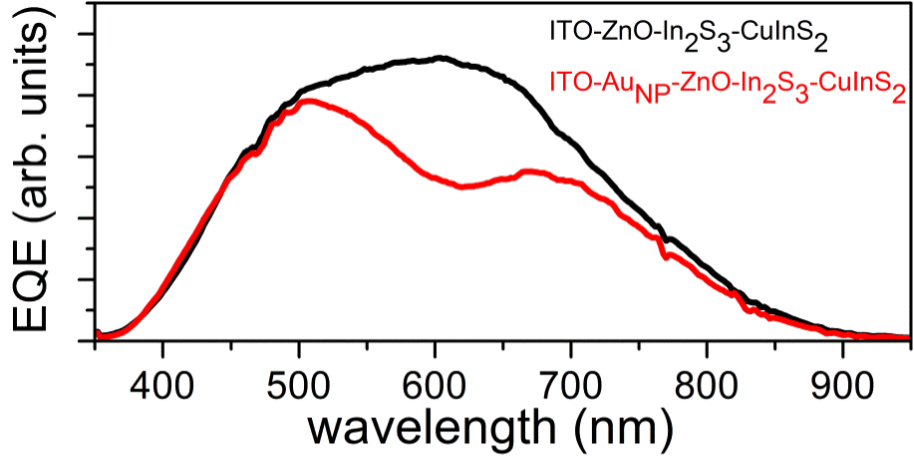
External quantum efficiency (EQE) of a plasmonic solar cell employing Au-NPs on top of an ITO layer. The EQE of the cells without the Au-NP layer is indicated with black lines. The volume of the solution of the Au-NP precursor was 2.5 mL.

**Figure 3:**
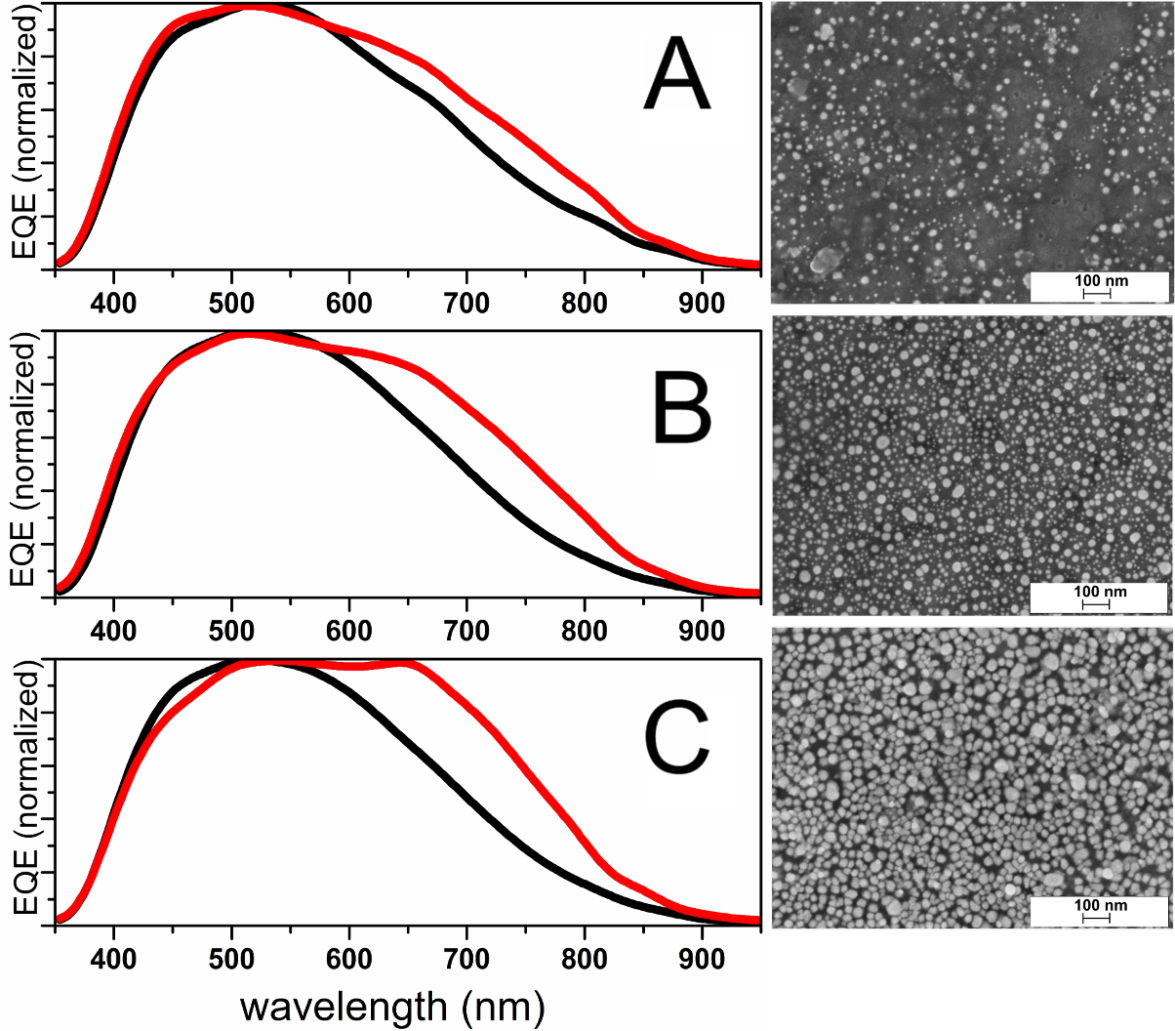
Left: External quantum efficiency (EQE) of ITO/ZnO/In_2_S_3_/CuInS_2_/Au-NP solar cells (red line) and EQE of the solar cells without the Au-NP layer (black lines). The volume of the Au-NP precursor solution was 2.5 mL (A), 5 mL (B) or 10 mL (C). Right: SEM images of the CuInS_2_/Au-NP surface aligned with the corresponding EQE. Note that the EQE graphs have been normalized to emphasize the relative gain of the EQE. The overall EQE decreased when 5 mL or 10 mL of the Au-NP precursor solution was sprayed.

A sketch of the solar cell is presented in Figure 1A for the design where the Au-NP layer follows the ITO layer and in Figure 1B for the configuration where the Au-NP layer follows the CuInS_2_ layer. The corresponding external quantum efficiency (EQE) spectra of the solar cells are presented in Figure 2 and Figure 3, respectively.

In Figure 2, it can be observed that the EQE of the solar cell with the ITO/Au-NP/ZnO/In_2_S_3_/CuInS_2_ configuration suffers losses in the region of 550–700 nm when compared to the reference solar cell without the Au-NP layer. The loss of EQE is attributed to the reflection of light from the Au-NP layer as evaluated from the reflectivity spectra of the cells (not shown).

Conversely, when the Au-NP layer is deposited on top of the CuInS_2_ layer (ITO/ZnO/In_2_S_3_/CuInS_2_/Au-NP), the EQE increases in the region of 600–850 nm (Figure 3A). When larger volumes of the Au-NP precursor solution are sprayed (up to 10 mL) onto the CuInS_2_, a further relative increase in the EQE is evident (Figure 3B,C) as compared to the reference spectra.

From these results, one can observe that the region of relative absorption gain was red-shifted with respect to the Au-NP on ITO configuration. The penetration depth of the optical radiation (in the region of 400–900 nm) remains roughly between 0.1 and 1 μm within the CuInS_2_ absorber [16]. Since the thickness of the CuInS_2_ is approximately 350 nm, a significant gain of absorption was not expected for wavelength regions with a low penetration depth. However, longer wavelengths penetrate deeper into the absorber (or are transmitted) and hence can fully utilize the presence the Au-NPs on the rear side of the absorber. In addition, Au is also an excellent reflector for wavelengths greater than 600 nm. Furthermore, an increase in the optical absorption can be expected in the red/infrared region for the sprayed CIS/Au-NP composite layers, as previously shown [9,17].

**Figure 4:**
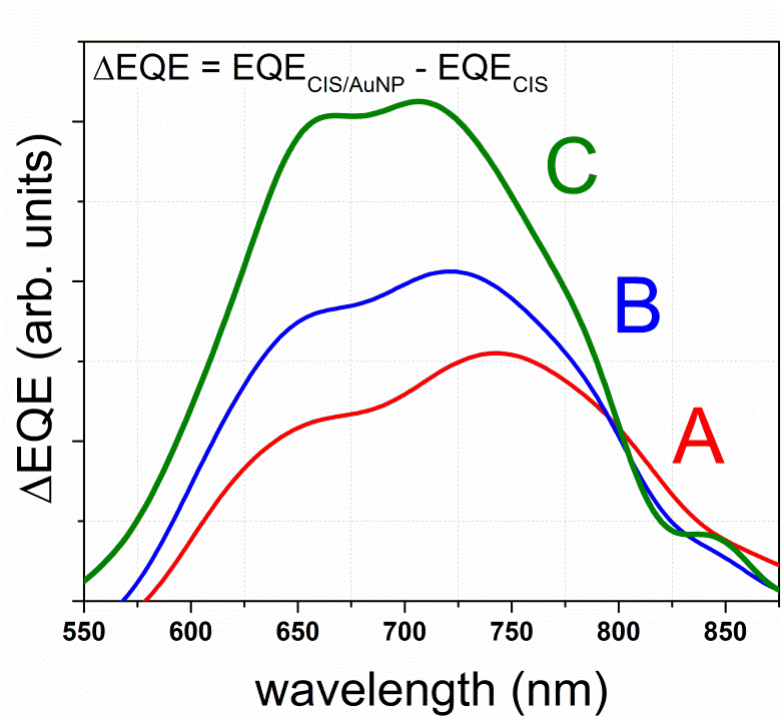
Spectra of the relative increase in ∆EQE for the ITO/ZnO/In_2_S_3_/CuInS_2_/Au-NP solar cells when compared to the EQE of the solar cells without the Au-NP layer. The ∆EQE curves A, B and C corresponds to the EQE data presented in Figure 3A, B and C, respectively.

The increase in the EQE is attributed to the increased CuInS_2_ coverage with Au-NPs when using a larger volume of the Au precursor solution [9]. The mean diameter of the individual spherical Au-NPs was between 20 and 60 nm for the 2.5–10 mL sprayed Au precursor solution, as evaluated from the SEM images (Figure 3). These values correspond well to those obtained in our previous study regarding Au-NPs produced by spray pyrolysis onto CuInS_2_ using HAuCl_4_ as a precursor [9]. However, Au-NP agglomerates of up to 200 nm can also be found, thus a size distribution of the of Au-NP agglomerates is also present. An increase in the particle size is likely to cause a red shift of the corresponding plasmon resonance, whereas a wide size distribution of Au-NPs and agglomerates is likely to cause a wide absorption band and a corresponding EQE gain due to the overlapping plasmon resonances [17]. To support this argument, details of the relative EQE gain (∆EQE = EQE_CIS/AuNP_ − EQE_CIS_) in the wavelength region of 550–900 nm are presented in Figure 4. Here, for the solar cell that uses 10 mL of Au precursor solution (curve C), at least three separate bands emerge centered at around 650, 710 and 850 nm.

Thus, the increase in the EQE in the red/infrared region (Figure 3) is likely due to the gain in optical absorptance induced by the surface plasmon resonance effect. For this effect to occur, the scattering medium must have a lower refractive index than that of the absorbing medium. As required, the refractive index of Au is in the range 1.5–0.2 [18] and that of CuInS_2_ is 3–2.6 [19] in the wavelength range of 400–900 nm.

It cannot be entirely excluded that charge transfer at the back contact region (graphite/gold/CuInS_2_) was improved with respect to the reference (graphite/CIS). However, resonant absorption peaks in the red/infrared region would not be expected to emerge (Figure 4) in the case of the charge transfer argument.

**Figure 5:**
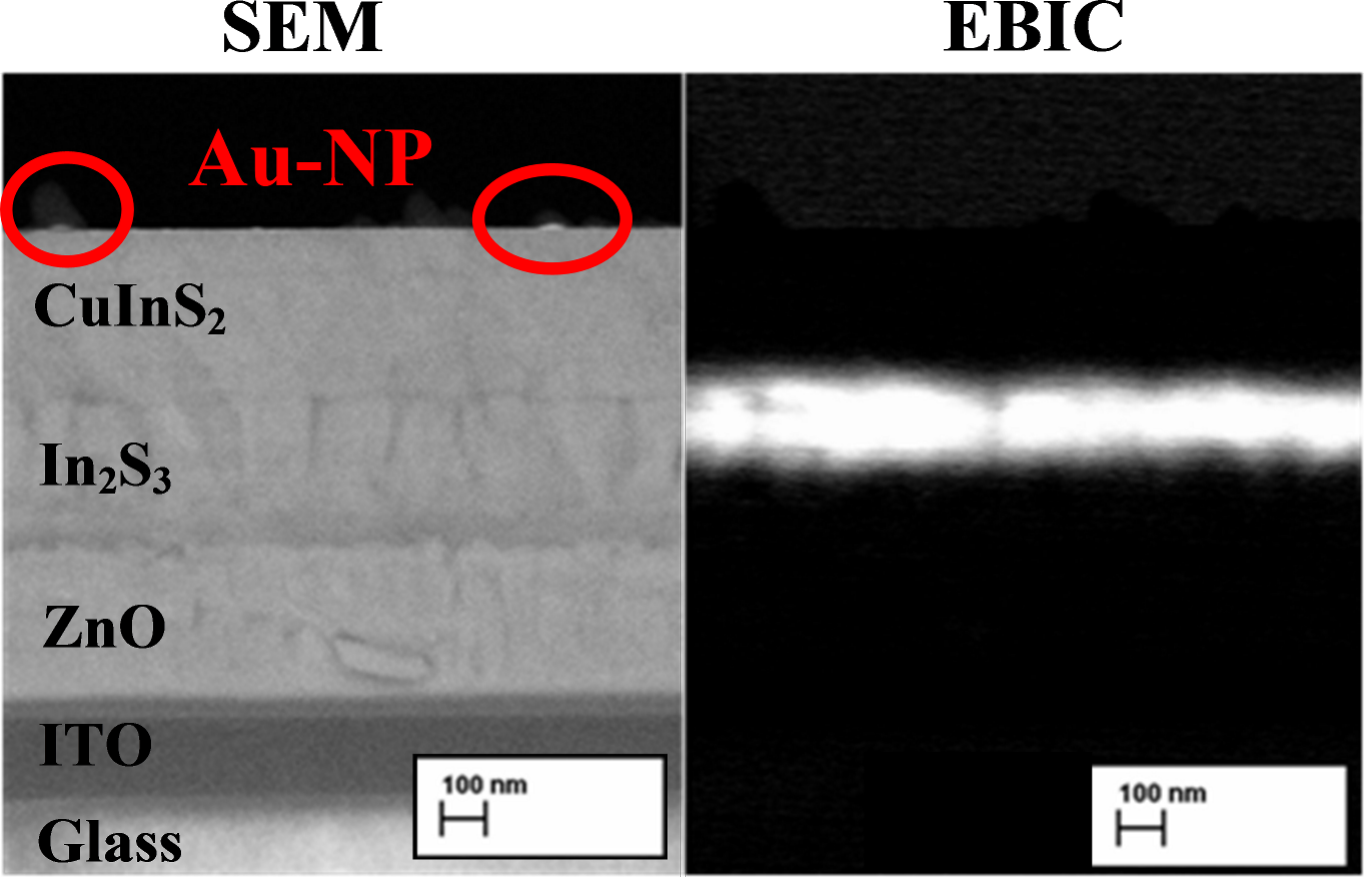
SEM image and EBIC image of the cross-section of the ZnO/In_2_S_3_/CuInS_2_/Au-NP solar cell prepared by spray pyrolysis on ITO/glass substrates. The Au-NP layer was deposited using 2.5 mL of the Au-precursor solution sprayed onto the CuInS_2_ layer at 260 °C. The images were acquired simultaneously from the same location on the sample.

For the solar cell prepared by spraying 2.5 mL of the Au precursor (Figure 3A) onto the CuInS_2_, the *V*_OC_ of the respective solar cell decreases from 448 to 414 mV (−8%), the current density, *J*_SC_, increases from 4.6 up to 7.5 mA/cm^2^ (+63%) and the FF decreases from 56 to 49% (−13%), as summarized in Table 1. The conversion efficiency η is proportional to *V*_OC_, and *J*_SC_ and FF and increase from 1.15 to 1.5% (+30%). Thus, the using of 2.5 mL of the precursor solution to deposit Au-NPs on the rear side of the solar cell is advantageous when compared to the deposition of Au-NPs onto the front of the cell. As an illustration, an SEM image and an EBIC image of the cross-section of the solar cell using 2.5 mL of Au precursor sprayed onto the CuInS_2_ is presented in Figure 5.

**Table 1:** Open-circuit voltage (*V*_OC_), short-circuit current (*J*_SC_), fill factor (FF) and light to electricity conversion efficiency (η) of ITO/ZnO/In_2_S_3_/CuInS_2_/Au-NP solar cell, evaluated from current–voltage measurements. The volume of the precursor solution for Au-NP was 2.5 mL. The EQE of the cell is presented in Figure 3A, the SEM image and EBIC image are presented in Figure 5.

	*V*_OC_ (mV)	*J*_sc_ (mA/cm^2^)	FF (%)	η (%)

ITO/ZnO/In_2_S_3_/CuInS_2_	448	4.6	56	1.15
ITO/ZnO/In_2_S_3_/CuInS_2_/Au_NP_	414	7.5	49	1.5

In contrast, when using larger volumes of Au-precursor solution (5 mL and 10 mL), the *V*_OC_, *J*_SC_ and FF all decrease (not shown). It was assumed that the decrease is a secondary effect since the CuInS_2_ layer will gradually dissolve/deteriorate when larger volumes of HAuCl_4_ are deposited [9]. To avoid the dissolution of CuInS_2_, deposition of presynthesized Au-NPs could be advantageous.

## Conclusion

Chemical spray pyrolysis (CSP) was employed to deposit ZnO/In_2_S_3_/CuInS_2_ solar cells that use an extremely thin CuInS_2_ absorber layer and an ITO substrate. Au-NPs as constituents of the solar cells were produced by spraying chloroauric acid (HAuCl_4_) onto the ITO or CuInS_2_ layer. The external quantum efficiency (EQE) of the solar cell decreased due to increased reflection of light due to the Au plasmon resonance in the region of 550–700 nm when the Au-NP layer was deposited onto the ITO layer. Conversely, the EQE of the solar cell increased when Au-NPs were deposited on top of the CuInS_2_ absorber material when a small volume of the HAuCl_4_ precursor was used. The increase in the EQE is due to increased absorption in region of 600–850 nm due to the Au-plasmon resonance. The increase in the absorption ability of the solar cell results in a relative increase of the conversion efficiency of the solar cells by 30% (from 1.15 to 1.5%) and a 63% increase (from 4.6 to 7.5 mA/cm^2^) in the short-circuit current of the solar cell. We have experimentally demonstrated that the deposition of a Au-NP layer on top of the CuInS_2_ absorber material can increase the absorption ability, the short-circuit current and the conversion efficiency of the ZnO/In_2_S_3_/CuInS_2_ solar cell, all prepared by an in-line, simple CSP method.
